# Correction: The Sec7 N-terminal regulatory domains facilitate membrane-proximal activation of the Arf1 GTPase

**DOI:** 10.7554/eLife.18204

**Published:** 2016-06-09

**Authors:** Brian C Richardson, Steve L Halaby, Margaret A Gustafson, J Christopher Fromme

Richardson BC, Halaby SL, Gustafson MA, Fromme JC. 2016. The Sec7 N-terminal regulatory domains facilitate membrane-proximal activation of the Arf1 GTPase. *eLife* **5**:e12411. doi: 10.7554/eLife.12411.

Published January 14, 2016

An error was detected in Figure 6—figure supplement 1. The original Figure highlighted residue Ser158 as the residue corresponding to the *sec7-1* mutation, when in fact the correct residue is Ser156. The corrected figure now highlights Ser156. The interpretations and conclusions are unaffected by this error. We note that the correct residue was highlighted in Figure 5A.

The corrected Figure 6—figure supplement 1 is shown here:

**Figure 1:**
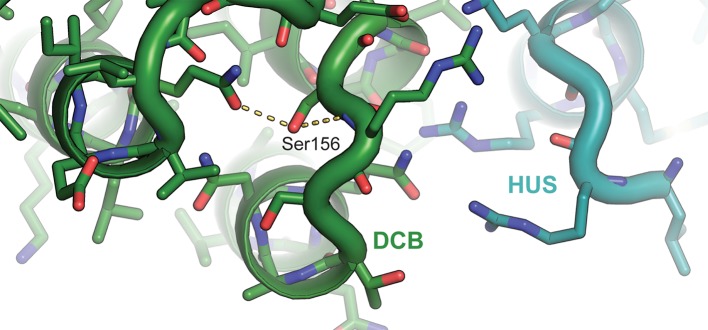

The originally published Figure 6—figure supplement 1 is also shown for reference:

**Figure 2:**
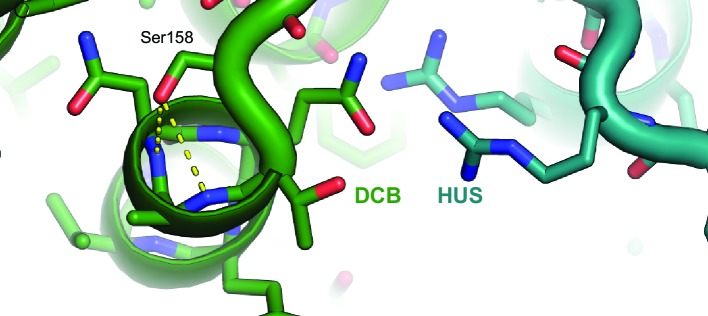

The corrected figure legend for Figure 6—figure supplement 1 is shown here:

**Atomic basis of the** ***sec7-1*** **phenotype**.

The temperature sensitive allele *sec7-1* represents an S402L mutation, corresponding to *T. terrestris* Sec7 residue S156. This serine stabilizes a loop near the interface between the DCB and HUS regions.

The originally published figure legend for Figure 6—figure supplement 1 is shown for reference:

**Atomic basis of the sec7-1 phenotype**.

The temperature sensitive allele *sec7-1* represents an S402L mutation, corresponding to *T. terrestris* Sec7 residue S158. This serine stabilizes a loop and N-terminal helical cap near the interface between the DCB and HUS regions.

The original text in the Results is shown for reference:

The established *sec7-1* temperature sensitive mutation (S402L in *S. cerevisiae*) (McDonold and Fromme, 2014; Novick et al., 1980) maps to a conserved serine at residue 158 in the α7–8 loop. As this serine sidechain provides helix-capping interactions (Figure 6—figure supplement 1), the leucine substitution arising from the *sec7-1* mutation likely perturbs the local structure of this region critical to forming the DCB/HUS interface.

These two sentences have been corrected to read:

The established *sec7-1* temperature sensitive mutation (S402L in *S. cerevisiae*) (McDonold and Fromme, 2014; Novick et al., 1980) maps to a conserved serine at residue 156 in the α7–8 loop (Figure 6—figure supplement 1). The leucine substitution arising from the *sec7-1* mutation likely perturbs the local structure of this region critical to forming the DCB/HUS interface.

The article has been corrected accordingly.

