# Supplementary material for: The Sec7 N-terminal regulatory domains facilitate membrane-proximal activation of the Arf1 GTPase
Source: eLife. 2016 Jan 14;5:e12411. doi: 10.7554/eLife.12411 (PMC4764562; doi:10.7554/eLife.12411)
Supplement: Supplementary file 1. — C. thermophilum, M. thermophila, and T. terrestris Sec7 genomic sequences, each containing a single annotated intron in the Sec7ΔC region, are aligned with annotated introns shown in lowercase. Conservation suggests that the T. terrestris intron should instead match that of the other two species; the intron assignment assumed for this work is highlighted in gray, and this correction to the construct was required for its expression (not shown). DOI: http://dx.doi.org/10.7554/eLife.12411.027 [file elife-12411-supp1.zip › Supplementary file 1.rtf]

                  810      820       830       840       850       860       870               
                 .|....|....|....|....|....|....|....|....|....|....|....|....|....|...
C. thermophilum  AAGTCAAGCCCATTAGGCCATCCGGGCGATCTGTCTCTGAGCAGAGCCTCAGTGAAAGTTCTCCGGAAGA 
M. thermophila   AGGTGAAACCCGTCAAGAGGGCGGCGCGGTCGGTGTCCGAGCAGAGTACCGCCGAGAGCTCCAACGAGGA 
T. terrestris    AGGTGAAACCCATGAAGAAGGCCTCGAGGTCGgtgtcggagcagagcctccaggaaagcccccaggacga 

                  880      890       900       910       920       930       940               
                 .|....|....|....|....|....|....|....|....|....|....|....|....|....|...
C. thermophilum  GTCGTCAGAAGCTTTGGATGCCGAAGACGAGGCCTACATCCGGGATGCTTACCTGGTGTTCCGTTCTTTC 
M. thermophila   TACGCCAGAATCCCTTGACGCCGAGGACGAAGCCTATATACGCGATGCTTACCTCGTTTTCCGGTCATTT 
T. terrestris    gaccccagagtcgttggacgccgaggacgaggcctacatacgggatgcgtacctcgtcttccgctcgttc 

                  950      960       970       980       990       1000      1010              
                 .|....|....|....|....|....|....|....|....|....|....|....|....|....|...
C. thermophilum  TGCAACCTCTCTACAAAGGTTCTGACACCGGATCAACTCTATGACCTTCGTGGCCAGGCCATGCGCTCCA 
M. thermophila   TGCAACCTGTCAACGAAGGTCCTCCCACCCGATCAACTTTACGATACCCGTGGACAGCCTATGCGCTCCA 
T. terrestris    tgcaatctgtcgaccaagatcttgccaccagaccagctatacgacctccgagggcaacccatgcgctcga 

                  1020     1030      1040      1050      1060      1070      1080              
                 .|....|....|....|....|....|....|....|....|....|....|....|....|....|...
C. thermophilum  AGCTTATCTCACTCCATATCATACACACCCTGCTTAACAACCACATCATTGTTTTCACGTCGCCCCTCTG 
M. thermophila   AGTTGATCTCGCTCCATCTCATTCACACACTGCTCAACAACCACATTACCGTCTTCACGTCACCGCTGTG 
T. terrestris    agctcatctcgctgcatctcattcacaccctgctcaacaaccatatcaccgtcttcacctcaccactctg 

                  1090     1100      1110      1120      1130      1140      1150              
                 .|....|....|....|....|....|....|....|....|....|....|....|....|....|...
C. thermophilum  CACGATCAGGAATACCAAAAATGGACAGTCCACACACTTTTTGCAGGCCATCAAGTATTATCTCTGCCTC 
M. thermophila   TACCATCAGGAACACGAAGAACAATGAGCCTACGAGTTTCCTGCAAGCTATCAAGTATTATCTTTGCCTG 
T. terrestris    cacgatcaggaacaccaagaataatgagcccacgaactttctgcaggccatcaagtactatctctgtctg 

                  1160     1170      1180      1190      1200      1210      1220              
                 .|....|....|....|....|....|....|....|....|....|....|....|....|....|...
C. thermophilum  AGCATCATTCGCAACGGTGCCAGCTCGGTAGACCGGGTGTTCGAAATTTGCTGCGAAATTTTCTGGCTGA 
M. thermophila   AGCATCACCCGCAATGGAGCAAGTTCTGTTGATAGGGTATTTGAAGTTTGTTGTGAAATCTTCTGGTTGA 
T. terrestris    agtatcacccgcaacggagcaagctcggtcgacagggtcttcgacatttgctgcgagattttctggttga 

                  1230     1240      1250      1260      1270      1280      1290              
                 .|....|....|....|....|....|....|....|....|....|....|....|....|....|...
C. thermophilum  TGCTGAAGTACATGAGAGCGCCATTCAAGgtcagtttgtgcgaaaaaaattggg---gccttcact---- 
M. thermophila   TGCTGAAGTACATGAGGTCCTCTTTCAAGgtcggt-------------cgcagg---ccccatactggca 
T. terrestris    tgctcaagtacatgaggtcatccttcaaggtcggt-------------agtggattcgccctttcttagg 

                  1300     1310      1320      1330      1340      1350      1360              
                 .|....|....|....|....|....|....|....|....|....|....|....|....|....|...
C. thermophilum  ---------------tgctgacacatgatcgcagAATGAAATTGCCGTATTCCTCAATGAAATCTACTTG 
M. thermophila   gctgcacaactgctaagctaatgtacaaacacagAACGAAATCGAGGTGTTCCTCAGCGAGATTTACCTT 
T. terrestris    cgtgcg-------ggagctgac-tgcaggtgcagAACGAGATCGAAGTCTTTTTGAACGAGATCTACCTC 

                  1370     1380   
                 .|....|....|....|.
C. thermophilum  GCCCTCCTAGCCCGGAAG 
M. thermophila   GCTCTCCTCGCACGGAGG 
T. terrestris    GCCCTTCTGGCCCGAAGA 
