# Supplementary material for: The Sec7 N-terminal regulatory domains facilitate membrane-proximal activation of the Arf1 GTPase
Source: eLife. 2016 Jan 14;5:e12411. doi: 10.7554/eLife.12411 (PMC4764562; doi:10.7554/eLife.12411)
Supplement: Supplementary file 2. — DOI: http://dx.doi.org/10.7554/eLife.12411.028 [file elife-12411-supp2.docx]

| **Plasmid ID** | **Insert** | **Species** | **Vector** | **Source** |
| --- | --- | --- | --- | --- |
| pBCR556 | 6His-Sec7(1-1888) | T. terrestris | pET28 | This study |
| pBCR558 | 6His-Sec7(1-1597) | T. terrestris | pET28 | This study |
| pBCR440 | 6His-Sec7(1-1030) | T. terrestris | pET28 | This study |
| pBCR441 | 6His-Sec7(1-818) | T. terrestris | pET28 | This study |
| pBCR445 | 6His-Sec7(1-492) | T. terrestris | pET28 | This study |
| pBCR444 | 6His-Sec7(1-458) | T. terrestris | pET28 | This study |
| pBCR557 | 6His-Sec7(616-818) | T. terrestris | pET28 | This study |
| pBCR314 | 6His-TEV-Sec7(203-2009) | S. cerevisiae | pFastBacHT | Richardson 2012 |
| pCM18 | 6His-TEV-Sec7(203-1799) | S. cerevisiae | pET28 | McDonold 2014 |
| pBCR389 | 6His-TEV-Sec7(203-1220) | S. cerevisiae | pET28 | Richardson 2012 |
| pMR02 | 6His-TEV-Sec7(203-1017) | S. cerevisiae | pET28 | Richardson 2012 |
| pMR01 | 6His-TEV-Sec7(203-756) | S. cerevisiae | pET28 | This study |
| pCM01 | 6His-TEV-Sec7(816-1017) | S. cerevisiae | pET28 | Richardson 2012 |
| pSH21 | 6His-TEV-Sec7(1915-2009) | S. cerevisiae | pET28 | This study |
| pBCR447 | 6His-TEV-Sec7(203-1017) D297A K301A F305A | S. cerevisiae | pET28 | This study |
| pBCR448 | 6His-TEV-Sec7(203-1017) D297A K301D F305D | S. cerevisiae | pET28 | This study |
| pBCR450 | 6His-TEV-Sec7(203-1017) Q365A R368A | S. cerevisiae | pET28 | This study |
| pBCR459 | 6His-TEV-Sec7(203-1017) R505A | S. cerevisiae | pET28 | This study |
| pBCR462 | 6His-TEV-Sec7(203-1017) K513D | S. cerevisiae | pET28 | This study |
| pBCR563 | 6His-TEV-Sec7(203-1017) N653A | S. cerevisiae | pET28 | This study |
| pBCR565 | 6His-TEV-Sec7(203-1017) C656A | S. cerevisiae | pET28 | This study |
| pBCR566 | 6His-TEV-Sec7(203-756) D297A K301A F305A | S. cerevisiae | pET28 | This study |
| pBCR567 | 6His-TEV-Sec7(203-756) D297A K301D F305D | S. cerevisiae | pET28 | This study |
| pBCR568 | 6His-TEV-Sec7(203-756) Q365A R368A | S. cerevisiae | pET28 | This study |
| pBCR570 | 6His-TEV-Sec7(203-756) R505A | S. cerevisiae | pET28 | This study |
| pBCR571 | 6His-TEV-Sec7(203-756) K513D | S. cerevisiae | pET28 | This study |
| pMG005 | Gea2(1-1459) | S. cerevisiae | pET28 | This study |
| pMG020 | Gea2(1-1196) | S. cerevisiae | pET28 | This study |
| pCF1299 | Gea2(1-782) | S. cerevisiae | pET28 | This study |
| pCF1084 | P_SEC7_-GFP-Sec7 | S. cerevisiae | pRS415 | Richardson 2012 |
| pCF1043 | P_SEC7_-Sec7 | S. cerevisiae | pRS416 | Richardson 2012 |
| pCF1157 | P_SEC7_-GFP-Sec7(1-1799) | S. cerevisiae | pRS415 | This study |
| pCF1141 | P_SEC7_-GFP-Sec7(1-1215) | S. cerevisiae | pRS415 | This study |
| pBCR583 | P_SEC7_-GFP-Sec7(203-2009) | S. cerevisiae | pRS415 | This study |
| pBCR506 | P_SEC7_-GFP-Sec7(203-2009) D297A K301A F305A | S. cerevisiae | pRS415 | This study |
| pBCR507 | P_SEC7_-GFP-Sec7(203-2009) Q365A R368A | S. cerevisiae | pRS415 | This study |
| pBCR508 | P_SEC7_-GFP-Sec7(203-2009) S402L | S. cerevisiae | pRS415 | This study |
| pBCR509 | P_SEC7_-GFP-Sec7(203-2009) S402W | S. cerevisiae | pRS415 | This study |
| pBCR510 | P_SEC7_-GFP-Sec7(203-2009) T416I | S. cerevisiae | pRS415 | This study |
| pBCR544' | P_SEC7_-GFP-Sec7(203-2009) Δ429-493 | S. cerevisiae | pRS415 | This study |
| pBCR511 | P_SEC7_-GFP-Sec7(203-2009) R505A | S. cerevisiae | pRS415 | This study |
| pBCR512 | P_SEC7_-GFP-Sec7(203-2009) R505D | S. cerevisiae | pRS415 | This study |
| pBCR545' | P_SEC7_-GFP-Sec7(203-2009) K513A | S. cerevisiae | pRS415 | This study |
| pBCR513 | P_SEC7_-GFP-Sec7(203-2009) K513D | S. cerevisiae | pRS415 | This study |
| pBCR514 | P_SEC7_-GFP-Sec7(203-2009) I597A R601A | S. cerevisiae | pRS415 | This study |
| pBCR515 | P_SEC7_-GFP-Sec7(203-2009) K606A | S. cerevisiae | pRS415 | This study |
| pBCR573 | P_SEC7_-GFP-Sec7(203-2009) N653A | S. cerevisiae | pRS415 | This study |
| pBCR574 | P_SEC7_-GFP-Sec7(203-2009) Y654A | S. cerevisiae | pRS415 | This study |
| pBCR575 | P_SEC7_-GFP-Sec7(203-2009) C656A | S. cerevisiae | pRS415 | This study |
| pBCR516 | P_SEC7_-GFP-Sec7 D297A K301D F305D | S. cerevisiae | pRS415 | This study |
| pBCR517 | P_SEC7_-GFP-Sec7 L364D R368D | S. cerevisiae | pRS415 | This study |
| pBCR537 | P_SEC7_-GFP-Sec7 Q365D R368D | S. cerevisiae | pRS415 | This study |
| pBCR538 | P_SEC7_-GFP-Sec7 R393A | S. cerevisiae | pRS415 | This study |
| pBCR520 | P_SEC7_-GFP-Sec7 R393D | S. cerevisiae | pRS415 | This study |
| pBCR539 | P_SEC7_-GFP-Sec7 N407A Q408A | S. cerevisiae | pRS415 | This study |
| pBCR542 | P_SEC7_-GFP-Sec7 T416A Q417A | S. cerevisiae | pRS415 | This study |
| pBCR540 | P_SEC7_-GFP-Sec7 T416D Q417D | S. cerevisiae | pRS415 | This study |
| pBCR535 | P_SEC7_-GFP-Sec7 K630A | S. cerevisiae | pRS415 | This study |
| pBCR526 | P_SEC7_-GFP-Sec7 D643A | S. cerevisiae | pRS415 | This study |
| pBCR527 | P_SEC7_-GFP-Sec7 I637A C641A D643A | S. cerevisiae | pRS415 | This study |
| pArf1 | Arf1 | S. cerevisiae | pET3 | Weiss 1989 |
| pBCR576 | L8K-Arf1 | S. cerevisiae | yArf1 | This study |
| pCF1053 | ΔN17-Arf1 | S. cerevisiae | pET28 | Richardson 2012 |
| pBCR551 | GST-Arf1(18-181) | S. cerevisiae | pGEX-6P | This study |
| pCF1184 | Arl1 | S. cerevisiae | pET23 | McDonold 2014 |
| pBCR435 | Arl1(14-183) | S. cerevisiae | pET28 | This study |
| pBCR550 | GST-Arl1(14-183) | S. cerevisiae | pGEX-6P | This study |
| pNMT1 | Nmt1 | S. cerevisiae | pCYC | Duronio 1990 |

| **Strain** | **Genome** | **Plasmid** | **GFP-Sec7 allele** | **Source** |
| --- | --- | --- | --- | --- |
| CFY409 | sec7∆::KanMX | pCF1043 |  | Richardson 2012 |
| CFY578 | suc2-∆9  Sec7-Mars::TRP1 |  |  | Jason MacGurn |
| CFY863 | sec7∆::KanMX  arf1∆::HIS3 | pCF1043 |  | Richardson 2012 |
| CFY866 |  | pCF1084 | WT | Richardson 2012 |
| CFY2078 |  | pBCR506 | D297A K301A F305A | This study |
| CFY2079 |  | pBCR516 | D297A K301D F305D | This study |
| CFY2080 |  | pBCR517 | L364D R368D | This study |
| CFY2081 |  | pBCR507 | Q365A R368A | This study |
| CFY2082 |  | pBCR537 | Q365D R368D | This study |
| CFY2083 |  | pBCR538 | R393A | This study |
| CFY2084 |  | pBCR520 | R393D | This study |
| CFY2085 |  | pBCR508 | S402L | This study |
| CFY2086 |  | pBCR509 | S402W | This study |
| CFY2087 |  | pBCR539 | N407A Q408A | This study |
| CFY2088 |  | pBCR540 | T416D Q417D | This study |
| CFY2089 |  | pBCR542 | T416A Q417A | This study |
| CFY2090 |  | pBCR510 | T416I | This study |
| CFY2091 |  | pBCR511 | R505A | This study |
| CFY2092 |  | pBCR512 | R505D | This study |
| CFY2093 |  | pBCR513 | K513D | This study |
| CFY2094 |  | pBCR514 | I597A R601A | This study |
| CFY2095 |  | pBCR515 | K606A | This study |
| CFY2096 |  | pBCR535 | K630A | This study |
| CFY2097 |  | pBCR527 | I637A C641A D643A | This study |
| CFY2098 |  | pBCR526 | D643A | This study |
| CFY2099 |  | pBCR544 | Δ429-493 | This study |
| CFY2100 |  | pBCR545 | K513A | This study |
