# Supplementary material for: The Sec7 N-terminal regulatory domains facilitate membrane-proximal activation of the Arf1 GTPase
Source: eLife. 2016 Jan 14;5:e12411. doi: 10.7554/eLife.12411 (PMC4764562; doi:10.7554/eLife.12411)
Supplement: Supplementary file 3. — Values for Arf1 trafficking calculations are provided. DOI: http://dx.doi.org/10.7554/eLife.12411.029 [file elife-12411-supp3.docx]

| Item | Value | Source |
| --- | --- | --- |
| Surface area of PM (A_PM_) | 78.5 μm^2^ | (Layton et al., 2011) |
| Surface area of vesicle (A_V_) | .031 μm^2^ | (Novick et al., 1980) |
| Doubling time (t_doubling_) | 6000 s |  |
| Vesicles needed for growth | .42 /s | = (A_PM­_ / A_V_) / t_doubling_ |
| Vesicles needed to counter endocytosis | .42 /s | (Layton et al., 2011) |
| Total exocytic vesicle rate | .84 /s |  |
| Arf1 density in COPI coat | 20,000 /μm^2^ | (Dodonova et al., 2015) |
| Arf1 proteins per vesicle | 620 | = density / area |
| Arf1 exocytosis rate | 520.8 /s | = protein count * vesicle rate |
| Yeast cell volume | 65 μm^3^ | (Layton et al., 2011) |
| Arf1 activation rate | 13 μM/s | = exocytosis rate / cell volume |
| Sec7 molecules per cell | 243 | (Chong et al., 2015) |
| [Sec7] | 6.2 μM | = Sec7 count / N_A_ / cell volume |
| Arf1 molecules per cell | 2065 | (Chong et al., 2015) |
| Fraction (mammalian) Arf1 GDP-bound | .66 | (Presley et al., 2002) |
| [Arf1-GDP] | 35 μM | = Arf1 count * GDP-bound / N_A_ / cell volume |
| Required rate constant | 60,000 /M/s | Arf1 activation rate = k * [Sec7] * [Arf1] |
